# Dog importation and changes in heartworm prevalence in Colorado 2013–2017

**DOI:** 10.1186/s13071-019-3473-0

**Published:** 2019-05-06

**Authors:** Jason Drake, Rudolph S. Parrish

**Affiliations:** Elanco Animal Health, 2500 Innovation Way, Greenfield, IN 46140 USA

**Keywords:** *Dirofilaria immitis*, Relocation, Animal rescue, Shelter, Animal welfare, USA

## Abstract

**Background:**

Animal welfare organizations, in order to increase dog adoptions and reduce euthanasia of homeless dogs, are transporting large numbers of dogs from state-to-state. Only approximately 1/3 of animal welfare organizations reportedly test for heartworm infection, treat, or provide heartworm prevention prior to transporting dogs. The reported prevalence of heartworms in dogs in animal shelters or rescued following natural disasters ranges between 14.6–48.8%. This analysis was conducted to evaluate the correlation between dog importation and heartworm prevalence in dogs in Colorado.

**Methods:**

Data were obtained from the Companion Animal Parasite Council (CAPC) interactive heartworm prevalence maps and the Colorado Department of Agriculture Pet Animal Care Facilities Program (PACFA) in order to analyze the relationship between dog importation and the changes in prevalence of heartworm (*Dirofilaria immitis*) in Colorado from 2013 to 2017.

**Results:**

The prevalence of heartworms in Colorado dogs has increased 67.5% between 2013–2017. From 2014 to 2017, more than 114,000 dogs were imported into Colorado by over 130 animal shelters and rescue organizations, which could represent approximately 9.5% of the total estimated 2017 Colorado dog population of nearly 1.2 million dogs. Three large Colorado-based organizations responded to requests for details regarding the originating states from which they received dogs. The majority of these dogs were apparently shipped to Colorado from states with higher heartworm prevalence. New Mexico represented the source of the greatest number of relocated dogs, accounting for just over 30%. Nearly half (49%) of the dogs relocated by these three organizations came from either Texas or Oklahoma.

**Conclusions:**

Animal welfare organizations and veterinarians should increase the testing and prevention of heartworm infections in dogs both prior to, and following, transportation from areas with high heartworm prevalence. Repeated testing is recommended due to the 6-month pre-patent-period associated with *D. immitis*. Veterinarians and pet owners should increase vigilance with heartworm testing and prevention, even in areas with historically low heartworm prevalence. Movement of dogs from highly endemic areas may increase the risks of local transmission.

## Background

Rescue organizations in the USA transport dogs interstate from areas where high numbers of stray animals are found into areas where these groups believe dogs have a better chance at being adopted. These organizations also provide important rescue and relocation services during severe weather emergencies like hurricanes and floods. A recent news report suggested nearly 30,000 dogs were imported into Colorado in a single year, primarily from the southern USA [1].

Infection with heartworm (*Dirofilaria immitis*) is relatively common in dogs from animal shelters and in dogs rescued from tornado and hurricane disasters in heartworm endemic areas. Heartworm disease, caused by *D. immitis*, can be difficult to detect prior to transporting dogs since the antigen and microfilaria tests utilized for detection depend upon the presence of adult worms, whereas infection can exist up to five months before adults are present. Testing dogs with infections less than six months of age will typically result in negative tests despite the presence of immature worms. Prevalence estimates in studies carried out during 2005–2018 range between 14.6–48.8% of dogs based on positive tests for *D. immitis* [2–6]. For example, a recent study in Mississippi including 363 dogs from 18 shelters in 17 Mississippi counties detected 34.4% and 22.6% samples positive for *D. immitis* antigen and microfilaria, respectively [6]. Another study showed 48.8% positive tests in 1958 dogs rescued and relocated from the Gulf Coast following the hurricanes in 2005 as tested by 141 animal welfare groups in 37 different states in the USA and Alberta, Canada [2]. A recent survey of rescue organizations reported that only 35.2% test, treat, or provide prevention to dogs for heartworms prior to transfer to a new facility [7].

In September 2018, Hurricane Florence struck the east coast of the USA. Just days prior to the hurricane making landfall, a dog rescuer made the news for gathering unadoptable dogs, including “the ones with heartworms”, from shelters, with plans to transport the dogs to other locations across the country [8]. Additional groups have also begun relocating dogs from North Carolina and South Carolina to other parts of the USA, including Connecticut, New York, Ohio, Pennsylvania and Washington D.C. [9–11]. Recent studies have identified an increasing prevalence of heartworm positive dogs across the USA, with some of the highest percentage increases occurring in states with historically low heartworm prevalence [12, 13]. This study was conducted to evaluate the changes in heartworm positive test rates in relation to the documented importation of dogs into Colorado during 2013–2017.

## Methods

The Companion Animal Parasite Council (CAPC) has compiled data on canine heartworm antigen testing based on antigen test results from two diagnostic laboratories (IDEXX Laboratories and ANTECH Diagnostics). These two labs conduct and provide results to CAPC for approximately 30% of all heartworm tests performed in the USA, with the results of more than 10 million heartworm tests reported in 2017 alone [14, 15]. The CAPC website provides monthly and yearly data on heartworm testing summarized by state and county *via* interactive maps from 2012 onward [15]. These data included the total number of tests reported, the number of heartworm-positive tests, and the percentage of positive tests for each county. The county-specific percentages of positive values are used here as estimates of the prevalence within the indicated counties in Colorado. Taken collectively, the total numbers of positive tests and numbers of tests conducted were used to estimate the overall prevalence of heartworm (HW) disease in the state. In particular, the yearly prevalence was estimated by dividing the total number of positive tests reported by the total number of tests conducted. Graphical displays corresponding to maps with counties delineated were created to display the yearly prevalence estimates on a county basis. These estimates were restricted to counties where at least 50 tests were reported, and these were calculated as the ratio of the number of positive tests to the number of tests reported for individual counties. No adjustment for county-level dog populations was incorporated. An underlying assumption was that the dogs that were tested constituted a representative sample of the dogs in the individual counties. In addition, the relative proportion of dogs tested was assumed to be similar among all counties reported. After compiling statewide estimates of canine heartworm prevalence by year, changes in yearly prevalence were calculated relative to 2013 as the baseline year. The CAPC website states that map data serve as a strong representation of area parasite activity [14]. CAPC also cautions that the data in the maps do not represent the total number of tests performed. Based upon the large number of samples obtained annually, we consider the data to be representative of the situation regarding canine HW testing and prevalence rates within Colorado. Total canine heartworm test results were obtained within the state of Colorado from the counties for which test results were available. With 2013 as the baseline year, changes in heartworm prevalence for each year were calculated from 2014 to 2017.

Statistical data regarding the transportation of dogs into Colorado by more than 130 different shelters and rescue organizations were obtained on an annual basis for 2014–2017 from the Colorado Department of Agriculture [16–19]. Using a linear regression model, the annual prevalence in Colorado was examined as a function of the number of dogs imported and years since 2013. Similarly, the change in percent prevalence in Colorado was examined as a function of the number of dogs imported, adjusted for the national prevalence.

Several of the largest Colorado organizations were contacted in order to gather more details regarding the originating states from where dogs were imported.

Population statistics including the number of households within Colorado were obtained from the US Census Bureau [20]. The number of dogs in Colorado was estimated using the following formula, provided by the American Veterinary Medical Association (AVMA) in their 2012 U.S. Pet Ownership & Demographics Sourcebook [21]: Number of dogs = 0.584 × total number of households in the community.

## Results

According to the United States Census Bureau, there were 2,051,616 households in Colorado 2012–2016 [20]. Using the formulas for estimating dog populations provided by the AVMA, there were approximately 1,198,143 dogs in Colorado in 2016.

According to the CAPC map data, approximately 10 million tests were performed in 2017 on an estimated population of 70 million dogs, representing 14.3% of dogs tested for heartworms in the USA. In Colorado 171,208 tests were performed in 2017 on an estimated population of 1.2 million dogs, representing 14.3% of dogs tested for heartworms.

Documentation related to importation of dogs from out-of-state sources was available from 37 of 64 Colorado counties (58%). For the remaining 27 counties (42%), no documentation related to dog importation was available.

For 28 of the 37 counties (76%) with evidence of out-of-state dog importation in one or more years during 2014–2017, there was also consistent availability of heartworm testing data within the CAPC maps for each year examined during 2013–2017. Only three of the 27 counties (11%) without documentation related to importation of out-of-state dogs had consistent availability of heartworm testing reported within the CAPC maps for each year.

From 2014 through 2017, more than 114,000 dogs were imported into the 37 counties in Colorado with 24,278, 28,147, 29,908 and 31,707 imported from out-of-state in 2014, 2015, 2016 and 2017, respectively. The number of dogs imported from out-of-state sources for 2014–2017 is reported in Table 1. Considering the estimated dog population of Colorado (1,198,143 dogs), documented imported dogs potentially represent 2.0–2.6% of the entire dog population of Colorado each year, assuming all imported dogs survive and have been properly reported by all shelters and animal welfare groups throughout the state. Three of the larger organizations in Colorado responded to requests for details regarding the source of imported dogs. The state representing the source of the greatest number of imported dogs was New Mexico, accounting for just over 30%. Nearly half (49%) of the dogs imported by these three groups came from either Texas or Oklahoma (Table 2).

**Table 1:** Dogs imported into 37 Colorado counties from out-of-state sources, 2014–2017

	2014	2015	2016	2017
Colorado (total)	24,278	28,147	29,908	31,707
Adams	1715	1381	2126	1749
Arapahoe	4322	3078	2356	2566
Archuleta	13	13	18	29
Boulder	2172	3026	2668	2960
Broomfield	160	837	476	376
Chaffee	4	0	39	48
Clear Creek	127	149	68	137
Costilla	46	18	0	0
Delta	414	536	1	0
Denver	957	1355	3159	2755
Douglas	734	1669	5702	5726
Eagle	35	17	6	55
El Paso	1447	2504	2942	4422
Elbert	172	166	124	171
Fremont	259	252	71	54
Garfield	141	102	126	176
Grand	388	253	201	191
Gunnison	83	100	1	44
Huerfano	0	65	280	0
Jefferson	3902	6040	4092	3908
Kit Carson	0	0	3	3
La Plata	365	303	258	20
Larimer	2080	679	2254	2731
Las Animas	736	825	1,042	495
Mesa	161	234	272	251
Montezuma	0	1	0	0
Morgan	8	9	7	18
Ouray	29	28	13	2
Park	9	70	36	328
Pitkin	141	155	53	47
Pueblo	83	21	31	220
Rio Blanco	78	113	0	0
Routt	0	0	48	10
Summit	331	340	127	98
Teller	339	37	94	420
Washington	0	0	0	145
Weld	2827	3771	1214	1552

**Table 2:** Top geographical sources of imported dogs

State	Count	Proportion (%)
NM	6488	30.32
TX	5638	26.35
OK	4883	22.82
CO	2194	10.25
KS	614	2.87
NE	459	2.15
LA	300	1.40

*Note*: 2017 imports reported by 3 of the larger organizations: Dumb Friends League, Humane Society of Pikes Peak and Humane Society of Boulder Valley

**Fig. 1:**
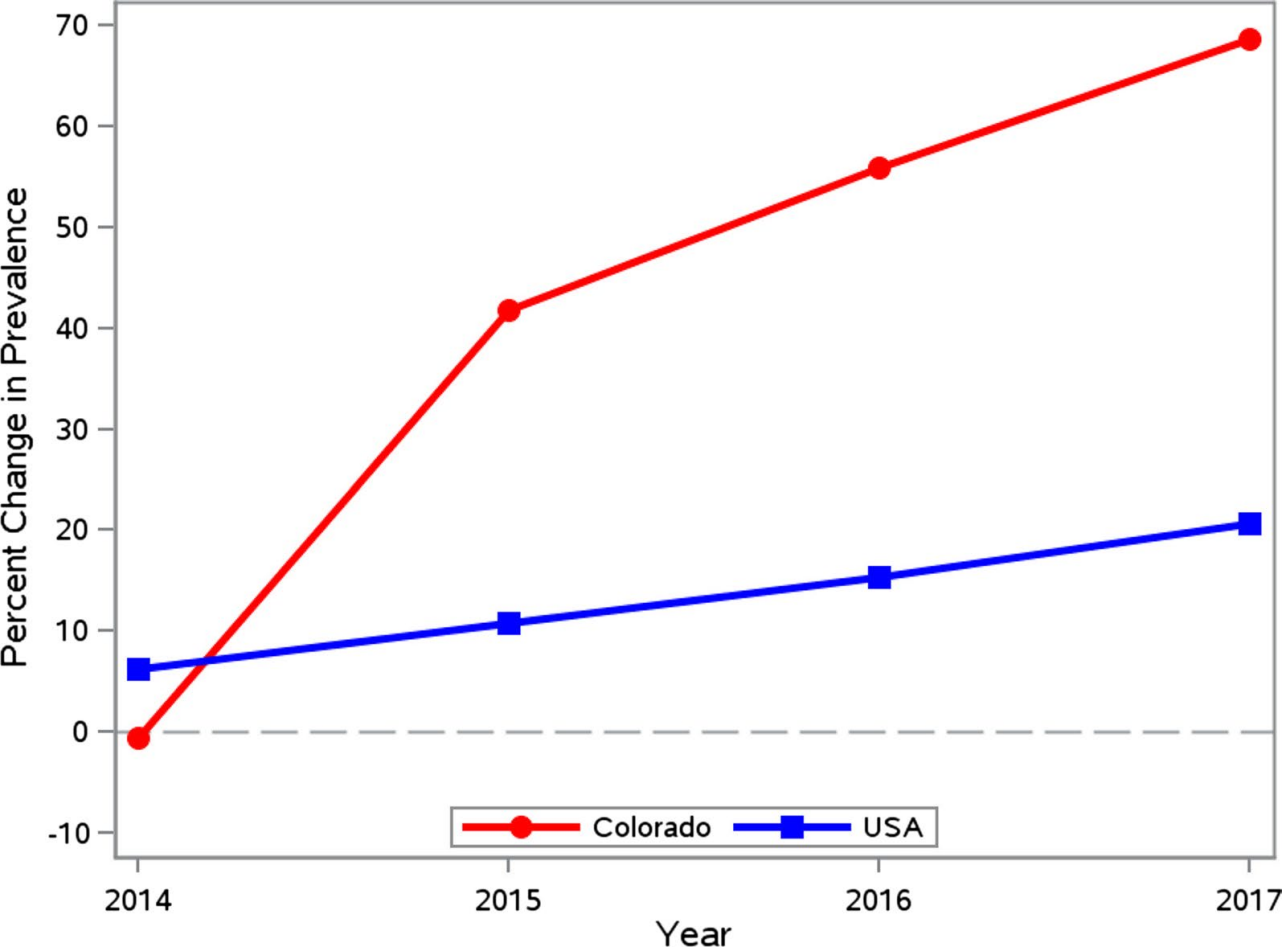
Percent change in heartworm prevalence since 2013 in Colorado (circles) and in the USA (squares)

**Fig. 2:**
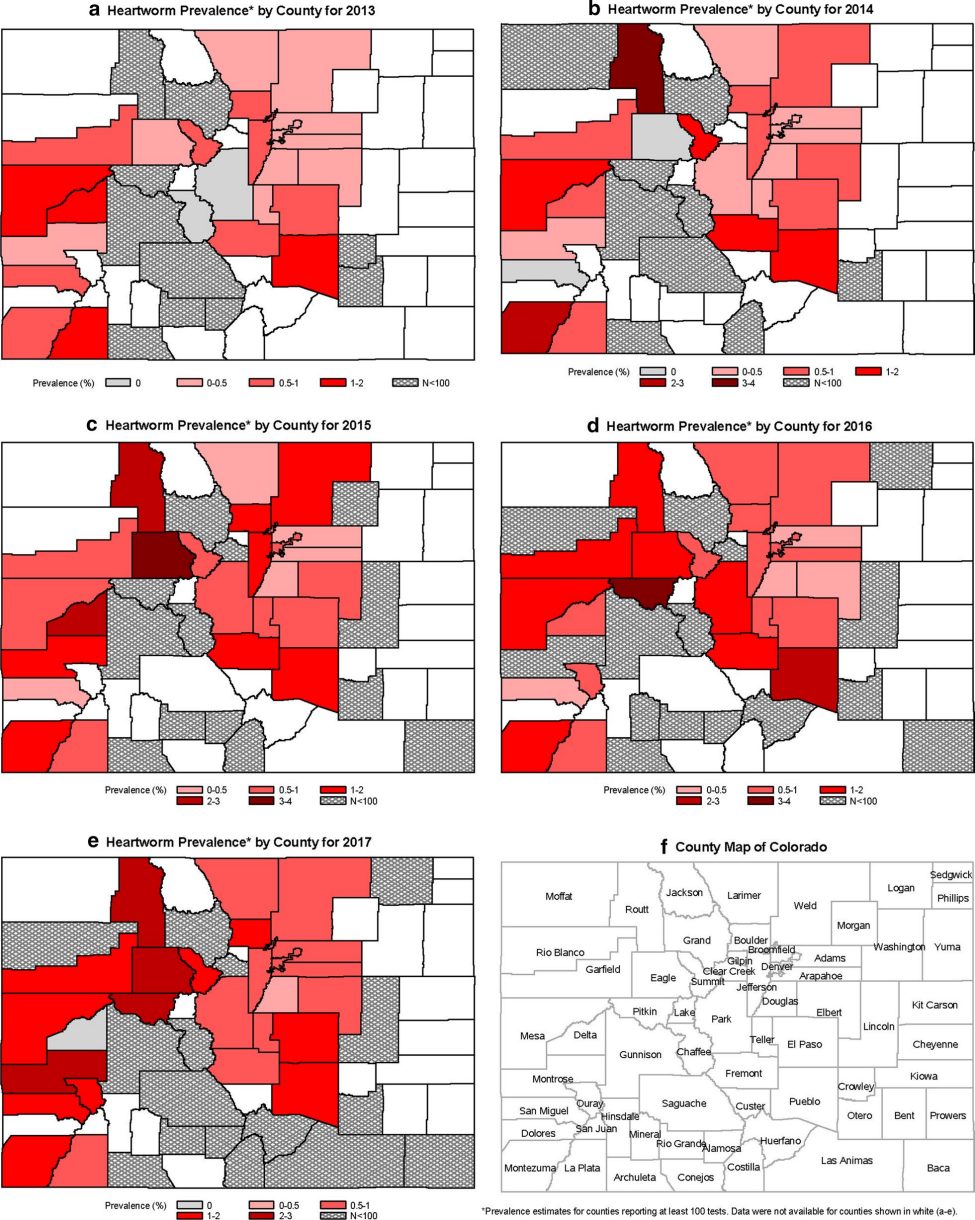
Heartworm prevalence for Colorado counties reporting at least 100 tests for years 2013–2017

**Fig. 3:**
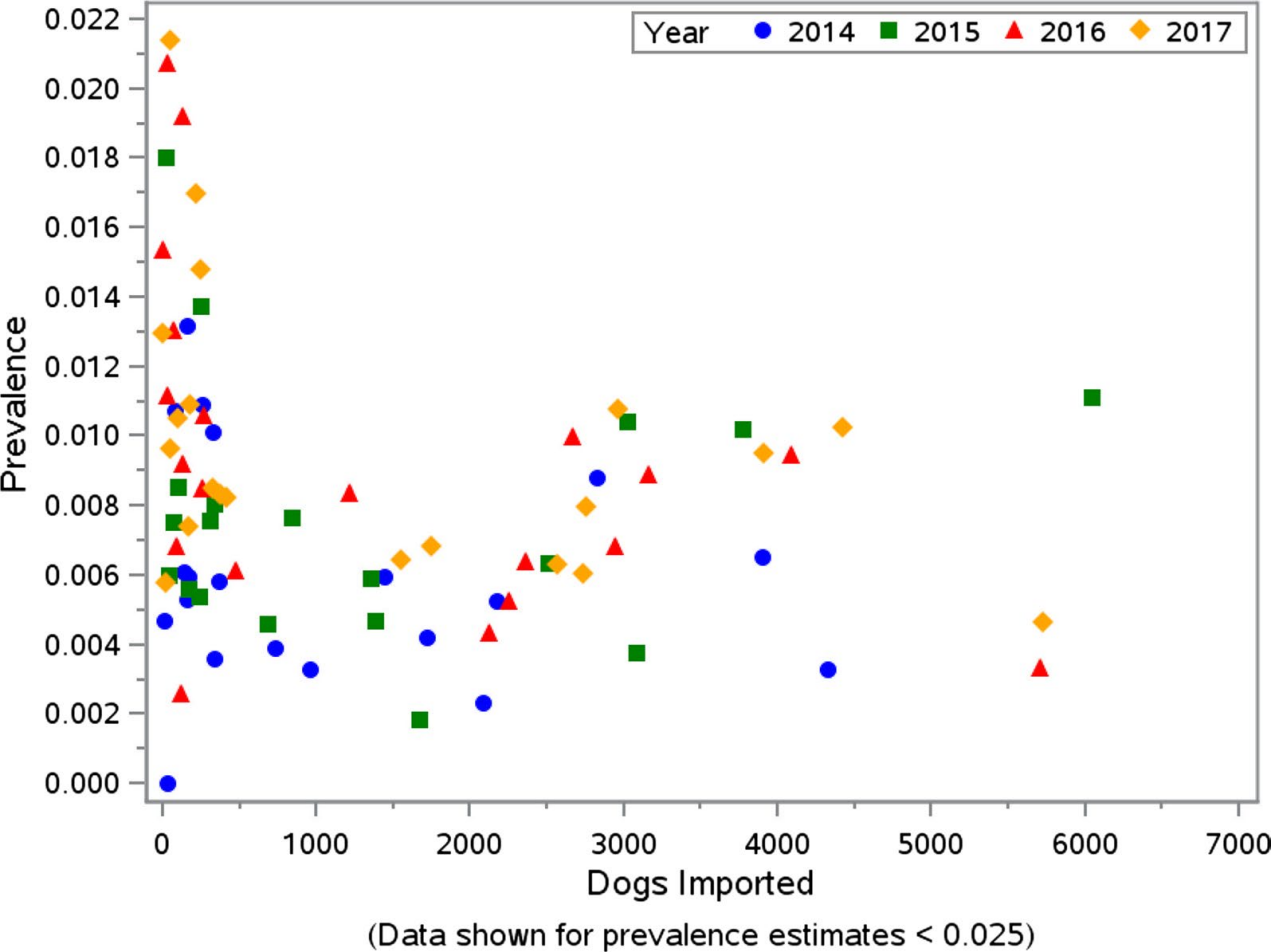
Heartworm prevalence in Colorado counties versus number of dogs imported for years 2014–2017. Data are shown for all counties with reported prevalence of less than 0.025 (2.5%)

**Fig. 4:**
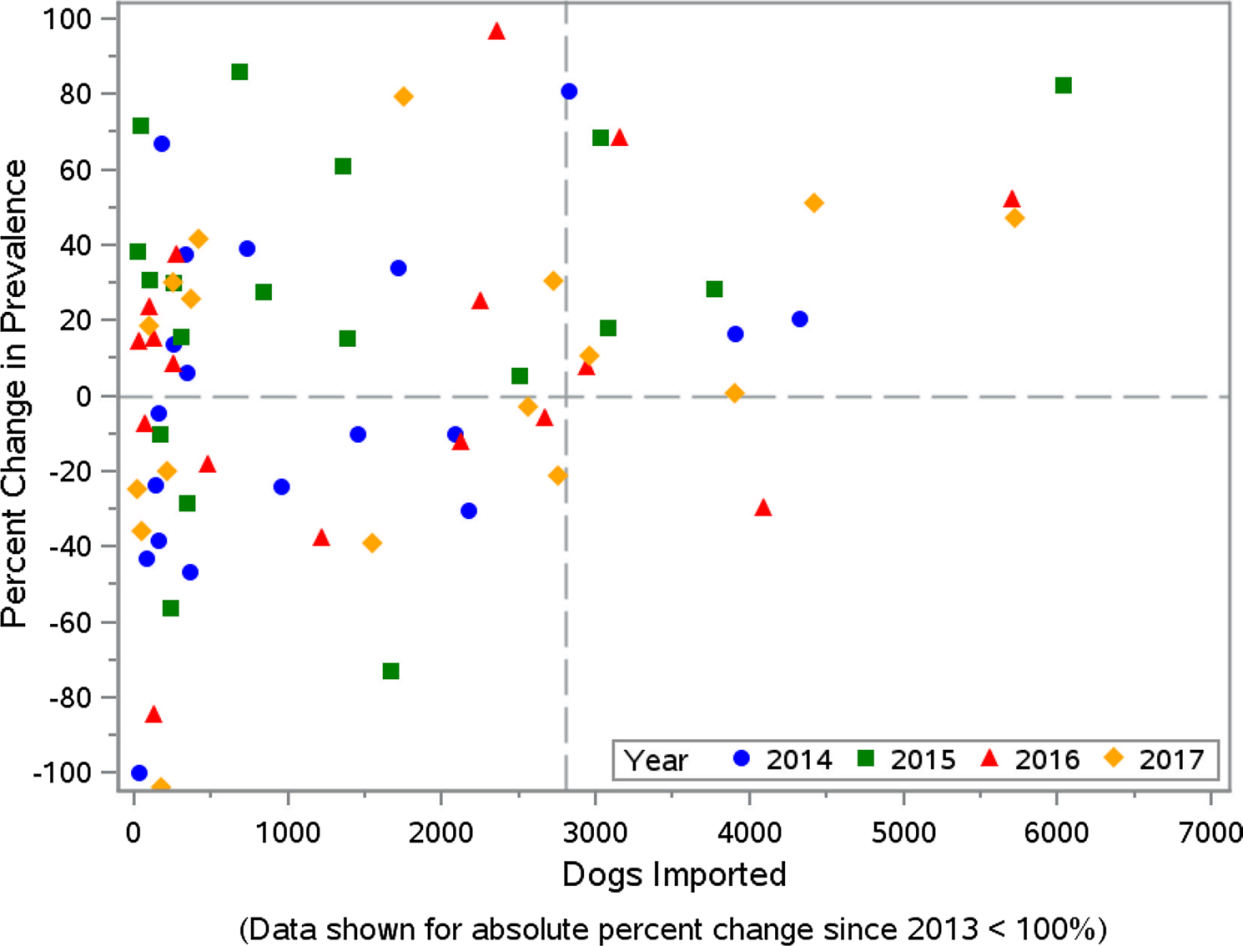
Percent change in heartworm prevalence since 2013 *versus* number of dogs imported for years 2014–2017. Data are shown for all counties with reported absolute percent change of less than 100%

During 2014–2017, the number of dogs imported into the 37 Colorado counties increased from 24,278 to 31,707 (30.6%) (Table 3). Based on the CAPC data, heartworm prevalence in Colorado in 2013 was 0.50%. This remained stable in 2014 at 0.50% and then rose annually in 2015, 2016 and 2017 with prevalence estimates of 0.71%, 0.78% and 0.84%, respectively (Table 4). The total number of tests reported, number of positive heartworm tests reported, and the percentage of dogs testing positive for heartworm infection from *Dirofilaria immitis* for each Colorado county are provided in Table 5. This increasing rate of heartworm prevalence was similar, albeit higher, to the increase nationwide; however, it represents an overall increase in heartworm prevalence for Colorado in 2017 of 69% when compared to 2013–2014 (Table 4), which is substantially higher when compared to the corresponding USA change of 21% (Fig. 1). The average annual increase in prevalence was estimated *via* linear regression to be 0.097 (SE 0.017) for Colorado and 0.056 (SE 0.002) for the USA (*R*^2^ = 0.92 and > 0.99, respectively). When considered as a function of the number of dogs imported annually, after adjusting for the US prevalence, prevalence in Colorado increased by 0.067 (SE 0.0003) for each thousand dogs imported (*P* = 0.0025). Maps of Colorado for the years 2013–2017 show increasing overall prevalence in most of the counties (Fig. 2). In viewing prevalence estimates for individual counties, where at least 200 tests were reported, Fig. 3 depicts a slight positive association between county-specific HW prevalence and number of dogs imported. Similarly, Fig. 4 shows a discernable increasing trend in percent change since 2013 in county-specific HW prevalence estimates. One point of 713% is not shown due to a low number of positive test results and scaling of the plot in Fig. 4. Notably, 14 of 15 times a county imported greater than 2800 dogs from out-of-state in one year, the county heartworm prevalence rose relative to 2013 levels (Fig. 4).

**Table 3:** Imported dogs and percentage of estimated total in Colorado (1,198,143 estimated dogs in state)

Year	Dogs imported^a^	Percent increase from prior year	Percent of total dogs
2014	24,278	–	2.026
2015	28,147	15.94	2.349
2016	29,908	6.26	2.496
2017	31,707	6.02	2.646

^a^Based on totals from 37 counties

**Table 4:** Heartworm prevalence in Colorado and the USA by year

Year	Colorado		USA	
	Prevalence (%)	Percent Change Since 2013	Prevalence (%)	Percent Change Since 2013
2013	0.498	–	1.11	–
2014	0.495	−0.6	1.18	6.3
2015	0.706	41.8	1.23	10.8
2016	0.776	55.8	1.28	15.3
2017	0.840	68.7	1.34	20.7

**Table 5:** Colorado canine heartworm prevalence by year in counties with documented dog importation from out of state [positive tests/total tests (%)]

	2013	2014	2015	2016	2017
Colorado (total)	610/122,585 (0.5)	641/129,433 (0.5)	1007/142,600 (0.71)	1208/155,636 (0.78)	1438/171,208 (0.84)
Adams^a^	15/4764 (0.31)	20/4742 (0.42)	35/7454 (0.47)	40/9264 (0.43)	60/8796 (0.68)
Arapahoe^a^	45/16,582 (0.27)	56/17,097 (0.33)	69/18,318 (0.38)	128/20,032 (0.64)	145/22,988 (0.63)
Archuleta	0/16 (0)	0/17 (0)	3/28 (10.71)	0/18 (0)	2/21 (9.52)
Boulder^a^	78/10,330 (0.76)	60/11,428 (0.53)	129/12,385 (1.04)	143/14,332 (1.0)	174/16,155 (1.08)
Broomfield^a^	6/703 (0.85)	8/1515 (0.53)	12/1568 (0.77)	15/2451 (0.61)	30/3612 (0.83)
Chaffee	0/1443 (0)	1/70 (1.43)	1/2 (50)	0/7 (0)	0/6 (0)
Clear Creek^a^	na	na	0/1 (0)	0/2 (0)	0/2 (0)
Costilla	na	0/12 (0)	0/4 (0)	na	0/2 (0)
Delta^a^	3/201 (1.49)	1/179 (0.56)	5/171 (2.92)	1/188 (0.53)	0/286 (0)
Denver^a^	95/22,042 (0.43)	81/24,739 (0.33)	154/26,046 (0.59)	237/26,737 (0.89)	236/29,669 (0.8)
Douglas^a^	31/11,037 (0.28)	45/11,514 (0.39)	23/12,386 (0.19)	47/14,123 (0.33)	70/15,065 (0.46)
Eagle^a^	1/221 (0.45)	0/215 (0)	9/279 (3.23)	5/326 (1.53)	5/234 (2.14)
El Paso^a^	92/13,849 (0.66)	87/14,592 (0.6)	102/16,121 (0.63)	114/16,661 (0.68)	180/17,598 (1.01)
Elbert^a^	2/562 (0.36)	4/673 (0.59)	4/716 (0.56)	2/776 (0.26)	7/946 (0.74)
Fremont^a^	12/1254 (0.96)	19/1745 (1.09)	16/1164 (1.37)	12/920 (1.3)	8/832 (0.96)
Garfield^a^	5/627 (0.8)	3/493 (0.61)	6/702 (0.85)	16/833 (1.92)	8/733 (1.09)
Grand^a^	1/11 (9.09)	0/7 (0)	3/13 (23.08)	3/16 (18.75)	1/10 (10)
Gunnison^a^	1/48 (2.08)	1/36 (2.78)	2/16 (12.5)	1/78 (1.28)	2/54 (3.7)
Huerfano^a^	na	na	na	0/6 (0)	0/51 (0)
Jefferson^a^	115/20,559 (0.56)	142/21,818 (0.65)	272/24,446 (1.11)	242/25,564 (0.95)	248/26,073 (0.95)
Kit Carson	na	na	na	na	na
La Plata^a^	10/912 (1.1)	5/858 (0.58)	8/1059 (0.76)	8/942 (0.85)	5/866 (0.58)
Larimer^a^	19/7282 (0.26)	18/7685 (0.23)	37/8061 (0.46)	45/8564 (0.53)	64/10,589 (0.6)
Las Animas^a^	na	na	na	na	0/1 (0)
Mesa^a^	15/1085 (1.38)	15/1138 (1.32)	8/1483 (0.54)	25/2357 (1.06)	44/2978 (1.48)
Montezuma	1/186 (0.54)	5/205 (2.44)	4/215 (1.86)	5/297 (1.68)	6/404 (1.49)
Morgan	na	na	0/1 (0)	na	na
Ouray	na	na	na	1/159 (0.63)	3/158 (1.9)
Park^a^	0/281 (0)	2/428 (0.47)	4/532 (0.75)	6/537 (1.12)	5/590 (0.85)
Pitkin^a^	0/23 (0)	0/11 (0)	1/35 (2.86)	7/232 (3.02)	16/774 (2.07)
Pueblo^a^	17/900 (1.89)	9/838 (1.07)	25/1388 (1.80)	30/1446 (2.07)	40/2358 (1.7)
Rio Blanco^a^	na	na	na	0/1 (0.0)	0/2 (0)
Routt	2/91 (2.2)	4/110 (3.64)	4/149 (2.68)	3/254 (1.18)	8/350 (2.29)
Summit^a^	12/1634 (0.73)	19/1878 (1.01)	18/2241 (0.80)	18/1963 (0.92)	16/1521 (1.05)
Teller^a^	1/296 (0.34)	1/279 (0.36)	3/499 (0.60)	6/881 (0.68)	11/1340 (0.82)
Weld^a^	20/4109 (0.49)	33/3751 (0.88)	44/4325 (1.02)	43/5156 (0.83)	37/5748 (0.64)

^a^More than 100 dogs imported from out-of-state 2013–2017

*Abbreviation*: na, not available

## Discussion

Colorado has historically been an area of relatively low heartworm prevalence. The prevalence of heartworms in Colorado rose at greater than 3-fold the national average during the observation period, rising from 0.5% in 2013 to 0.84% in 2017, an increase of 67.5% in just four years During that same time period, the prevalence of heartworms in the USA has risen by 20.7%.

Based upon the number of heartworm tests reported and the calculated dog population, the rate of heartworm testing in Colorado (14.3%) appears to be equal to the national rate of testing in the USA (14.3%). The total heartworm tests performed in the USA or Colorado are not available. ANTECH Diagnostics, Inc. and IDEXX Laboratories, Inc. test results reported through CAPC maps represent about a third of the tests performed.

Only about 35% of animal shelters and rescue organizations tests, treat or provide heartworm prevention prior to transporting dogs. Complicating the situation even further, it takes 6–7 months for a dog to become heartworm antigen- or microfilaria-positive following infection. With more than 114,000 dogs imported into Colorado from 2014 to 2017 from out-of-state by over 130 animal shelters and rescue groups, pet owners and veterinarians in these areas, or similar areas with historically low heartworm prevalence, may not be adequately aware of the parasitic risks posed by imported dogs.

Data from three of the larger organizations importing dogs in Colorado showed nearly half of the dogs originate from Oklahoma and Texas (Table 2). According to the CAPC maps for 2017, 2.2% of dogs in Oklahoma and 3.45% of dogs in Texas tested positive for heartworm [22, 23]. The prevalence of heartworm in Oklahoma and Texas was 3–4 fold higher than the prevalence of heartworm in Colorado in 2017 [24].

Colorado counties with consistent reporting of heartworm testing data also tended to be counties with documentation of dog importation. A total of 27 counties have no importation data reported. This may be due to the lack of shelters and animal welfare groups importing dogs in these counties or may be due to a lack of reporting by groups in these counties. This may be related to overall county population, easy access to veterinary services and the number of animal rescue groups located within these counties. Additional studies may be needed to evaluate the reasons for availability of data between counties.

The prevalence of heartworm infections in dogs is increasing in Colorado and may be underestimated. In addition to the dogs testing positive for heartworm antigen, some heartworm-infected dogs will be missed if only antigen testing is performed as some dogs may have immature heartworm infections that the antigen tests will not detect, and some dogs will be heartworm microfilaria-positive, yet antigen-negative [25, 26].

Animal shelters and rescue organizations are not effectively addressing heartworm infections prior to the movement of infected dogs. Many of these organizations also reported moving dogs within the state of Colorado, which could account for heartworm prevalence increases in counties without a group reporting importations [16–19]. According to three of these Colorado-based organizations, just over 10% of the dogs they receive are from other sources in Colorado (Table 2). Pet owners and veterinarians in areas with historically low heartworm prevalence need to be extra vigilant when caring for and adopting dogs with an unknown heartworm preventive history especially when imported from areas with potentially higher parasitic infection risks.

At least three species of mosquitoes that are competent vectors of heartworm, including *Aedes vexans*, *Culex pipiens* and *Anopheles freeborni*, can be found in Colorado [27]. *Aedes vexans* is the most commonly trapped mosquito species in Colorado [28] and can migrate long distances towards urban areas and lights [27]. Presence of these competent vectors will allow for continued local transmission of heartworm within Colorado.

The AVMA reports 1.6 dogs per dog-owning household [21]. Dogs living in a home with a heartworm-infected dog may be at much higher risk of acquiring heartworms, especially when considering the impact of a heartworm positive dog on the infection rate of mosquitoes, which can be as high as 73.7% inside a kennel [29]. Dogs imported from areas with a higher prevalence of heartworms should be tested, started on heartworm prevention, and regularly re-tested to rule out the presence of pre-existing infestations.

## Conclusions

Veterinarians in Colorado should no longer base heartworm testing and prevention recommendations on only historic heartworm risks and prevalence. Heartworm prevalence in Colorado rose 67.5% from 2013–2017. Veterinarians and pet owners in areas with historically low heartworm prevalence should reevaluate the increasing risks posed by heartworms and should follow the testing and prevention guidelines provided by organizations like the American Heartworm Society and the Companion Animal Parasite Council [26, 30]. While animal shelters and other animal welfare organizations provide important rescue and relocation services, relocated dogs may be introducing higher risks of parasitic diseases. Repeated heartworm testing and movement restrictions should be considered for dogs originating from states with a high prevalence of *D. immitis* in order to reduce the spread of parasitic diseases. Imported dogs initially testing negative for the presence of adult heartworms should be re-tested six months later as antigen tests are not reliable at detecting immature infections. Further research regarding the heartworm status of imported dogs could help confirm the association of increasing heartworm prevalence and dog importation.

## Abbreviations

AVMA: American Veterinary Medical Association
CAPC: Companion Animal Parasite Council
HW: heartworm
PACFA: Pet Animal Care Facilities Act
SE: standard error

## References

[1] Simpson K. Tens of thousands of out-of-state shelter dogs find welcoming destination—and salvation—in Colorado. Denver Post. 25 February 2018. https://www.denverpost.com/2018/02/25/out-of-state-shelter-dogs-welcome-colorado/. Accessed 23 Aug 2018.

[2] Levy JK, Edinboro CH, Glotfelty CS, Dingman PA, West AL, Kirkland-Cady KD (2007). Seroprevalence of *Dirofilaria immitis*, feline leukemia virus, and feline immunodeficiency virus infection among dogs and cats exported from the 2005 Gulf Coast hurricane disaster area. JAVMA.

[3] Tzipory N, Crawford PC, Levy JK (2010). Prevalence of *Dirofilaria immitis*, *Ehrlichia canis* and *Borrelia burgdorferi* in pet dogs, racing greyhounds, and shelter dogs in Florida. Vet Parasitol.

[4] Levy JK, Lappin MR, Glaser AL, Birkenheuer AJ, Anderson TC, Edinboro CH (2011). Prevalence of infectious diseases in cats and dogs rescued following Hurricane Katrina. JAVMA.

[5] Barrett AW, Little SE (2016). Vector-borne infections in tornado-displaced and owner-relinquished dogs in Oklahoma, USA. Vector Borne Zoonotic Dis.

[6] Donnett U, Hubbard K, Woodruff K, Varela-Stokes A (2018). Prevalence of canine heartworm infection in Mississippi animal shelters. Vet Parasitol.

[7] Simmons Kaitlyn, Hoffman Christy (2016). Dogs on the Move: Factors Impacting Animal Shelter and Rescue Organizations’ Decisions to Accept Dogs from Distant Locations. Animals.

[8] Flynn M. Noah’s Ark except it’s a school bus: truck driver rescues 64 dogs and cats from floods of Hurricane Florence. Washington Post. 17 September 2018. https://www.washingtonpost.com/news/morning-mix/wp/2018/09/17/noahs-ark-except-its-a-school-bus-truck-driver-rescues-64-dogs-and-cats-from-floods-of-hurricane-florence/?noredirect=on&utm_term=.c5cb32998209. Accessed 17 Sep 2018.

[9] Woytack C. Friendship APL takes in 6 dogs moved out of S.C. ahead of Florence. The Chronicle-Telegram. 17 September 2018. http://www.chroniclet.com/Local-News/2018/09/17/Friendship-APL-takes-in-6-dogs-moved-out-of-S-C-ahead-of-Florence.html. Accessed 18 Sep 2018.

[10] Guza M. Dogs from shelters in Florenceʼs path arrive in Pittsburgh. Trib Total Media. 17 September 2018. https://triblive.com/local/allegheny/14089351-74/dogs-from-shelters-in-florences-path-arrive-in-pittsburgh. Accessed 19 Sep 2018.

[11] Lang M. These cats and dogs were moved to safety as Hurricane Florence bears down on the East Coast. The Washington Post. 12 September 2018. https://www.washingtonpost.com/local/these-cats-and-dogs-were-moved-to-safety-as-hurricane-florence-bears-down-on-the-east-coast/2018/09/12/5cf37e70-b6ac-11e8-94eb-3bd52dfe917b_story.html?utm_term=.0d64aef474d6. Accessed 18 Sep 2018.

[12] Drake J, Heinz-Loomer C, Rotenberry A, Wiseman S. Increasing incidence of *Dirofilaria immitis* in dogs in USA 2013–2017. In: Proceedings, 63rd Annual AAVP Meeting, Denver, CO, USA; 2018.

[13] Drake J, Wiseman S (2018). Increasing incidence of *Dirofilaria immitis* in dogs in the USA with focus on the southeast region 2013–2016. Parasites Vectors.

[14] Companion Animal Parasite Council—CAPC maps “understanding the map data”. https://www.capcvet.org/maps. Accessed 17 Sep 2018.

[15] Companion Animal Parasite Council—canine heartworm map data. https://www.capcvet.org/maps#2016/all/heartworm-canine/dog/united-states/colorado/. Accessed 24 Aug 2018.

[16] Colorado Department of Agriculture Pet Animal Care Facilities Program (PACFA). 2014—animal shelter & rescue annual reporting numbers. https://data.colorado.gov/Agriculture/2014-Animal-Shelter-Rescue-Annual-Reporting-Number/gyi8-8sq8. Accessed 23 Aug 2018.

[17] Colorado Department of Agriculture Pet Animal Care Facilities Program (PACFA). 2015—shelter and rescue statistics. https://data.colorado.gov/Agriculture/2015-Shelter-And-Rescue-Statistics/773d-wy2e. Accessed 23 Aug 2018.

[18] Colorado Department of Agriculture Pet Animal Care Facilities Program (PACFA). 2016—shelter and rescue statistics. https://data.colorado.gov/Agriculture/2016-Shelter-And-Rescue-Statistics/m8vm-brgw. Accessed 23 Aug 2018.

[19] Colorado Department of Agriculture Pet Animal Care Facilities Program (PACFA). 2017—shelter and rescue statistics. https://data.colorado.gov/Agriculture/2017-Individual-Shelter-And-Rescue-Statistics/uhi6-hddy. Accessed 17 Sep 2018.

[20] US Census Bureau. QuickFacts Colorado—population estimates July 1, 2017. https://www.census.gov/quickfacts/fact/table/co#viewtop. Accessed 24 Aug 2018.

[21] American Veterinary Medical Association. U.S. pet ownership statistics. https://www.avma.org/KB/Resources/Statistics/Pages/Market-research-statistics-US-pet-ownership.aspx. Accessed 24 Aug 2018.

[22] Companion Animal Parasite Council—canine heartworm map data. https://www.capcvet.org/maps#2017/all/heartworm-canine/dog/united-states/oklahoma/. Accessed 29 Nov 2018.

[23] Companion Animal Parasite Council - canine heartworm map data. https://www.capcvet.org/maps#2017/all/heartworm-canine/dog/united-states/texas/. Accessed 29 Nov 2018.

[24] Companion Animal Parasite Council—canine heartworm map data. https://www.capcvet.org/maps#2017/all/heartworm-canine/dog/united-states/colorado/. Accessed 29 Nov 2018.

[25] Little SE, Munzing C, Heise SR, Allen KE, Starkey LA, Johnson EM, Meinkoth J, Reichard MV (2014). Pre-treatment with heat facilitates detection of antigen of *Dirofilaria immitis* in canine samples. Vet Parasitol.

[26] American Heartworm Society. Prevention, diagnosis and management of heartworm (*Dirofilaria immitis*) infection in dogs. https://www.heartwormsociety.org/veterinary-resources/american-heartworm-society-guidelines. Accessed 25 Jan 2019.

[27] Ledesma N, Harrington L (2011). Mosquito vectors of dog heartworm in the United States: vector status and factors influencing transmission efficiency. Top Companion Anim Med.

[28] Eisen L, Bolling BG, Blair CD, Beaty BJ, Moore CG (2008). Mosquito species richness, composition, and abundance along habitat-climate-elevation gradients in the northern Colorado Front Range. J Med Entomol.

[29] Mckay T, Bianco T, Rhodes L, Barnett S (2013). Prevalence of *Dirofilaria immitis* (Nematoda: Filarioidea) in mosquitoes from northeast Arkansas, the United States. J Med Entomol.

[30] Companion Animal Parasite Council. CAPC guidelines—heartworm. https://capcvet.org/guidelines/heartworm/. Accessed 25 Jan 2019.

